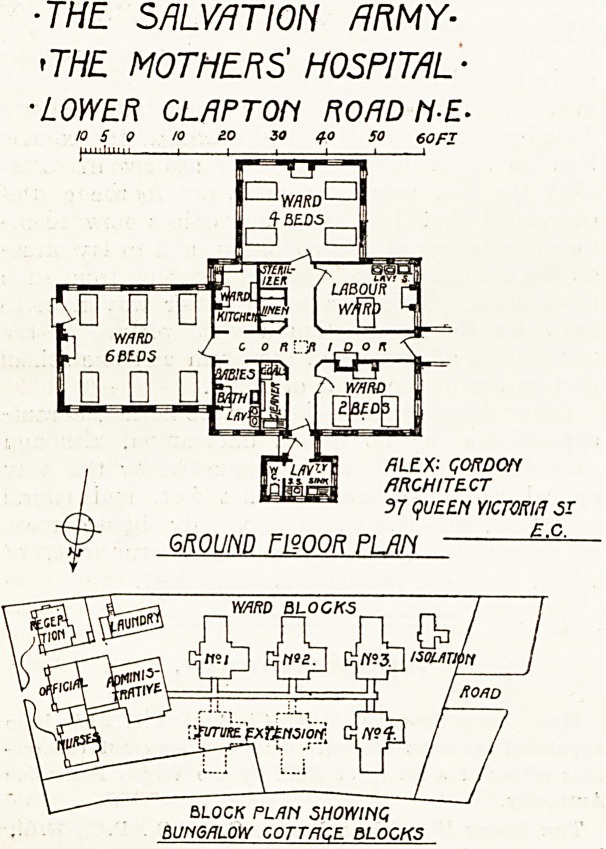# The Salvation Army Mothers' Hospital

**Published:** 1913-10-25

**Authors:** 


### THE SALVATION ARMY MOTHERS' HOSPITAL.

On the 18th inst. H.R.H. Princess Louise inaugurated this important branch of the Salvation Army's social work in the East End of London.

The site, some $2\frac{3}{4}$ acres in extent, seems quite an ideal one for the purpose. Fronting the Lower Clapton Road are three pairs of semi-detached houses which have been altered and adapted with additional buildings to provide administrative offices, an admission block, a nurses' house, and a laundry. On the land behind six separate ward blocks, of which four have been completed, and a small isolation block are planned. These blocks

GROUND FLOOR PLAN

BLOCK PLAN SHOWING BUNGALOW COTTAGE BLOCKS

are all one storey only in height and are connected with the main administrative block by corridors. The ward blocks are so disposed that every ward will have ample access of sunlight and air.

The division of the hospital into separate blocks or bungalows, each containing twelve patients, has been adopted with a view to the classification of the patients, a most desirable object. Thus one bungalow will be for married women, one for unmarried, one for special cases (presumably complications), and one it is hoped will be for Jewish mothers.

A detailed plan of one bungalow is given, by which it will be seen that there are three wards containing respectively six, four, and two beds, a labour room, ward kitchen, baby washing room, sterilising room, linen cupboard, and cleaners' room, with a sanitary block provided with the orthodox " cut off " lobby.

Judging from the plan the corridors would seem to be insufficiently lighted, but possibly in the actual building this may not be the case. The baby washing room might with advantage have been increased in size. Two lavatory basins close to a window seem hardly a suitable provision for washing newly born babies. The size of the linen store will, we fear, prove inadequate for the very large amount of linen required for twelve beds. Provision ought also to have been made for a nurses' lavatory. Apart from these points the block is admirably designed for the purpose in view.

The isolation block contains accommodation for one patient and two nurses.

The hospital was designed by Mr. Alexander Gordon with the co-operation of Dr. Mackintosh, M.B., C.V.O., of the Western Infirmary, Glasgow.

## Figures and Tables

**Figure f1:**